# *HLA* genotypes and cold medicine-induced Stevens–Johnson syndrome/toxic epidermal necrolysis with severe ocular complications: a systematic review and meta-analysis

**DOI:** 10.1038/s41598-020-67610-5

**Published:** 2020-06-29

**Authors:** Wimonchat Tangamornsuksan, Sirikan Chanprasert, Phenphechaya Nadee, Saowalak Rungruang, Natnaree Meesilsat, Mayumi Ueta, Manupat Lohitnavy

**Affiliations:** 1 Faculty of Medicine and Public Health, HRH Princess Chulabhorn College of Medical Science, Chulabhorn Royal Academy, Bangkok, Thailand; 2 Department of Dental Public Health, Sirindhorn College of Public Health, Phitsanulok, Thailand; 3 Integrative Cardiovascular Research Unit, Faculty of Medical Science, Naresuan University, Phitsanulok, Thailand; 4 Department of Physiology, Faculty of Medical Science, Naresuan University, Phitsanulok, Thailand; 5 Department of Frontier Medical Science and Technology for Ophthalmology, Kyoto Prefectural University of Medicine, Kyoto, Japan; 6 Center of Excellence for Environmental Health and Toxicology, Faculty of Pharmaceutical Sciences, Naresuan University, Phitsanulok, 65000 Thailand; 7 Pharmacokinetic Research Unit, Faculty of Pharmaceutical Sciences, Naresuan University, Phitsanulok, Thailand; 8 Department of Pharmacy Practice, Faculty of Pharmaceutical Sciences, Naresuan University, Phitsanulok, Thailand

**Keywords:** Risk factors, Outcomes research

## Abstract

Serious cutaneous adverse drug reactions [i.e., SJS/TEN with severe ocular complications (SOC)] associated with cold medicine (CM) were reported in several studies. To assess the risks of CM-induced SJS/TEN with SOC, systematic review and meta-analysis were employed. Studies investigating associations between *HLA* genotypes and CM-induced SJS/TEN with SOC were systematically searched in PubMed, Scopus and the Cochrane Library. Overall odds ratios (ORs) with 95% CIs were calculated using a random-effects model to determine these associations. An initial search of the databases identified 24,011 articles, of which 6 studies met the inclusion criteria. In total from all studies, associations between 81 different HLA genotypes and CM-induced SJS/TEN with SOC (i.e., 22 different *HLA-A* genotypes, 40 different *HLA-B* genotypes and 19 different *HLA-C* genotypes) were investigated. Risk factors to develop SJS/TEN with SOC in patients who used CM were identified from our meta-analysis. HLA-A*0206 (OR = 3.90; 95% CI = 1.96–7.77), HLA-A*3303 (OR = 2.28; 95% CI = 1.31–3.97), HLA-B*4403 (OR = 3.27; 95% CI = 1.52–7.03) and HLA-C*0501 (OR = 2.55; 95% CI = 1.19–5.44) were associated with CM-induced SJS/TEN with SOC. With our results demonstrating a significant association between using of CMs and the severe ADR, a genetic testing can be helpful. However, the CMs are commonly used as an over-the-counter drug in practically almost of people in populations worldwide, the genetic screening prior to use of the CMs might not be cost-effective. Nonetheless, for people with a family history of developing the ADRs with a possible involvement of CMs, a genetic screening may be beneficial.

## Introduction

Stevens–Johnson syndrome (SJS) and toxic epidermal necrolysis (TEN) represent a scale of severity of one of the most severe cutaneous hypersensitivity reactions^1,2^. These conditions are characterized by acute blisters arising on purple macules on the skin and at least two sites of mucosal membranes such as the ocular surface, oral cavity, and genitals. The initial manifestations include fever, stinging in the eyes, and pain in swallowing, and later developing cutaneous lesions can involve entire body^3,4^. Incidence of SJS/TEN is extremely low (0.4–6 cases per million persons per year)^5,6^, however, mortality rates are as high as 1–5% for SJS and 25–35% for TEN^2,5^. In addition, approximately 50%-88% of patients with SJS/TEN develop ocular complications, potentially leading to destructive results such as corneal damage and loss of vision^7–9^. The mechanisms underlying the onset of SJS/TEN have not been fully established^2^. Although the involvement of immune mechanisms, altered drug metabolism and infections such as *Mycoplasma pneumoniae* and herpes viruses have been suggested^2,10,11^.

Common cold is one of the most common illnesses affecting millions of people around the world^12^. Cold medicine (CM) such as non-steroidal anti-inflammatory drugs (NSAIDS) and other multi-ingredient formulations are widely used for relieving its symptoms. There were some studies reporting serious cutaneous adverse drug reactions (i.e., SJS/TEN with severe ocular complications (SOC) in patients with a history of taking the cold medicines^13,14^. Few of recent studies demonstrated statistically significant associations between adverse drug reactions and genetic predisposition specific alleles of human leukocyte antigen (HLAs) genes^15–21^. *HLA* present antigenic peptides to passing T-cells and distinct genotypes have been previously shown to control development of autoimmune disease^22^. Cold medicine-induced Stevens–Johnson Syndrome/Toxic epidermal necrolysis with severe ocular complications (CM-induced SJS/TEN with SOC) has been associated with various *HLA* genotypes were also reported several epidemiological studies^14,23,24^. A strong association between HLA-B*4403 and CM-induced SJS/TEN with SOC were reported in Japanese, Indian and Brazilian populations^23,24^. Whereas HLA-A*0206 was associated only in Japanese and Korean populations, but not among the Indian population^23,24^. These findings might be related to the prevalence of individual susceptibility alleles in each population. Therefore, to consolidate these findings in populations, systematic review and meta-analysis techniques were employed to determine associations between certain *HLA* genotypes and CM-induced SJS/TEN with SOC in different populations.

## Methods

### Search strategy and selection criteria

PubMed, Scopus and the Cochrane Library were systematically searched from their inception until March 2019 using keyword combinations or synonyms for “*HLA* genotypes” and “SJS/TEN” without drugs or study design restrictions. Only English language and human studies were included. Additional studies were retrieved from bibliographies of the included articles.

Reviewers (SC, SR, PN, NM and WT) independently screened titles and/or abstracts for relevance followed by full-text article assessments for inclusion. Studies were included if: (1) HLA genotype associations were investigated for CM-induced SJS/TEN with SOC; (2) all patients received CM before HLA genotype screening, and; (3) sufficient data for calculating the frequency of HLA genotype carriers were reported. When studies shared the same population, the one reporting most complete would be selected. Where data were insufficient for meta-analysis, additional data would be sought from corresponding authors of the selected studies.

Reviewers (SC and WT) extracted data by study design, eligibility criteria, definition, and diagnostic criteria for cases and controls, patient demographics and type of CM exposure, the HLA genotyping technique and Hardy–Weinberg equilibrium (HWE) information^25^. The genotype frequencies were examined by the HWE to determine whether the patients from the selected studies were representative of the population^26,27^. To assess the quality of the selected studies, the Newcastle–Ottawa scale (NOS) was employed^28^. All disagreements throughout were resolved by discussion between the reviewers until consensus was made.

### Data analysis

The included studies demonstrating an association between *HLA* genotypes and CM-induced SJS/TEN with SOC were characterized and summarized based on the most recent data. The overall odds ratios (ORs) with 95% confidence intervals (CIs) were calculated to determine association between *HLA* genotypes and CM-induced SJS/TEN with SOC. All analyses were performed using the DerSimonian and Laird method under a random-effects model^29^. The analyses were also performed separately on studies using different *HLA* genotypes and different race/ethnicity. Statistical heterogeneity was assessed via the Q-statistics and I-squared tests^30^. *P* values ≤ 0.10 indicated heterogeneity between studies. I-squared values of 25%, 50%, 75% denote a low, moderate, and high degree of heterogeneity across studies^31^. All statistical analyses were performed using the R program (version 3.4.0) (R foundation for statistical computing, 2017).

## Results

### Search strategy and selection criteria

**Figure 1:**
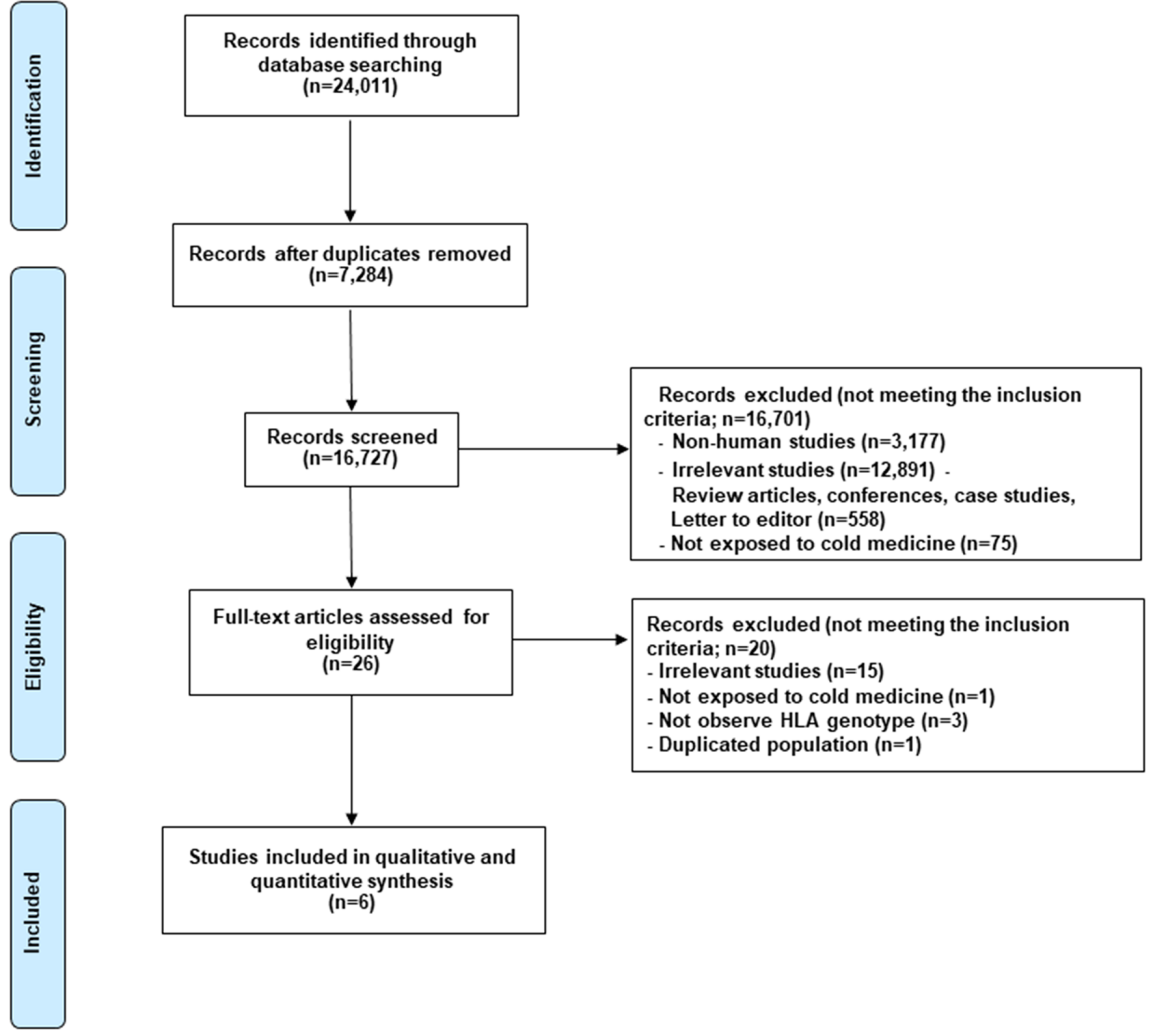
PRISMA flow diagram of *HLA* genotypes and CM-induced SJS/TEN with SOC.

A PRISMA flow diagram is depicted in Fig. 1. The initial search from the databases identified 24,011 articles. After duplicate records were removed, 16,727 articles were first screened on the basis of title and/or abstract to determine the eligibility. We then excluded 16,701 articles for the following reasons: (1) They were not human studies (3,177 articles); (2) Studies did not meet the inclusion criteria (12,891 articles); (3) They were review articles, case reports, letters to editor, commentaries or conference abstracts (558 articles); and; (4) The patients in the studies did not receive CM (75 articles);

There were 26 articles which met the full inclusion criteria. After full-text assessment, some studies had overlapped period of data collection and the same *HLA* genotype. Therefore, correct data were sought from corresponding authors of the selected studies. Notably, some populations of included studies were duplicated^24,32^. Therefore, studies reporting most/complete data and patients were selected. In addition, there were 2 studies sharing information of certain HLAs^24^. After all, 20 out of 26 were excluded and reasons leading exclusion are summarized in Fig. 1. There were 6 studies for further meta-analysis. No additional articles were identified via review of the bibliographies of the included studies^23,24,33–36^.

### Study characteristics and quality assessment

All of the included studies were case–control studies^23,24,33–36^. Characteristics and demographical information of the patients were summarized in Table 1 and Supplemental Table 1. In total of 302 patients with CM-induced SJS/TEN with SOC and 1,107 healthy controls (no reviews of history taking of CM but it can be assumed that these populations have taken them because they are generally used world-wide) were included in our systematic review and meta-analysis^23,24,33–36^. Mean ages of included patients were 34.4 years and 38.4 years in cases and healthy controls, respectively^23,24,33–36^. Male subjects made up 38.41% (116 of 302) of cases and 33.60% (372 of 1,107) of healthy controls^23,24,33–36^.

**Table 1:** Characteristics of studies included in the meta-analysis.

Author (year)	Study design	Participant ethnicity	Number of participant		*HLA* genotypes		
			Case (n)	Control (n)	*HLA-A* genotypes	*HLA-B* genotypes	*HLA-C* genotypes
Ueta et al. (2014)^23^	Case control	Japanese	151 (Group1 KPUM: n = 131; Group2 NIHS: n = 20)	639 (Group1 KPUM: n = 419; Group2 NIHS: n = 220)	HLA-A*0206, HLA-A*0301, HLA-A*1101, HLA-A*2402	HLA-B*1301, HLA-B*1501, HLA-B*4402, HLA-B*4403, HLA-B*4601, HLA-B*5201, HLA-B*5401	HLA-C*0304, HLA-C*0501, HLA-C*1202
Ueta et al. (2014)^24^	Case control	Indian	20	55	HLA-A*0206	HLA-B* 4403	HLA-C*0701
		Brazilian	39 (Ethnicity: Pardo, n = 19; White, n = 15; Black, n = 3; White/Indian, n = 1; White/Black/Indian, n = 1)	134 (Ethnicity: Pardo, n = 66; White, n = 62; Black, n = 4, Indian/White, n = 2)			
		Korean	31	90			
Kannabiran et al. (2017)^33^	Case control	Indian	80	50	HLA-A*0101, HLA-A*0211, HLA-A*0301, HLA-A*1101, HLA-A*2402,HLA-A*2601, HLA-A*3201,HLA-A*3303, HLA-A*6801	HLA-B*0705, HLA-B*1502, HLA-B*3501, HLA-B*3503, HLA-B*4006, HLA-B*4403, HLA-B*5101, HLA-B*5201, HLA-B*5701	HLA-C*0102, HLA-C*0401, HLA-C*0602, HLA-C*0701, HLA-C*0702, HLA-C*0801, HLA-C*1202, HLA-C*1502
Wakamatsu et al. (2017)^34^	Case control	Brazilian	39 (Ethnicity: European, n = 16; Pardo^a^, n = 19)	133 (Ethnicity: European, n = 61; Pardo, n = 66)	HLA-A*0101, HLA-A*0201, HLA-A*0202, HLA-A*0205, HLA-A*0206, HLA-A*0301, HLA-A*1101, HLA-A*2402, HLA-A*2601, HLA-A*2902, HLA-A*3001, HLA-A*3002, HLA-A*3101, HLA-A*3201, HLA-A*3303, HLA-A*3402, HLA-A*3601,HLA-A*6601, HLA-A*6801,HLA-A*6802, HLA-A*7401	HLA-B*0702, HLA-B*0801, HLA-B*1302, HLA-B*1401, HLA-B*1510, HLA-B*1801, HLA-B*2705, HLA-B*3501, HLA-B*3502, HLA-B*3503, HLA-B*3508, HLA-B*3801, HLA-B*3903, HLA-B*4001, HLA-B*4002, HLA-B*4101, HLA-B*4201, HLA-B*4402, HLA-B*4405, HLA-B*4901, HLA-B*5001,HLA-B*5101, HLA-B*5201,HLA-B*5301, HLA-B*5501,HLA-B*5601, HLA-B*5701,HLA-B*5703, HLA-B*5801,HLA-B*5803	HLA-C*0102, HLA-C*0202, HLA-C*0302, HLA-C*0303, HLA-C*0304, HLA-C*1203, HLA-C*0401, HLA-C*0501, HLA-C*0602, HLA-C*0701, HLA-C*0702, HLA-C*0704, HLA-C*0802, HLA-C*1203, HLA-C*1402, HLA-C*1502, HLA-C*1601, HLA-C*1701
Jongkhajornpong et al. (2018)^35^	Case control	Thai	71	119	HLA-A*2402, HLA-A*3303	HLA-B*2704, HLA-B*4401, HLA-B*4403	HLA-C*0701
Jun et al. (2019)^36^	Case control	Korean	40	120	HLA-A*0206		HLA-C*0304, HLA-C*0503

*HLA* human leukocyte antigen, *KPUM* Kyoto Prefectural University of Medicine, *NIHS* National Institute of Health Science.

^a^Pardo is a commonly used term to refer to Brazilians of mixed ethnic ancestries, typically white Brazilians and Afro-Brazilians.

All of the included studies were conducted in Asian (one studies in Japanese populations,^23^ 2 studies in Korean populations,^24,36^ 2 studies in Indian populations^24,33^ and another study in Thai population^35^) and South American populations (2 studies in Brazilian populations^24,34^).

Four studies defined CM as multi-ingredient CM including NSAIDs.^23,24,34,35^ All of the included studies investigated associations between 81 different *HLA genotypes* and CM-induced SJS/TEN with SOC (i.e., 22 different *HLA-A* genotype, 40 different *HLA-B* genotypes and 19 different *HLA-C* genotypes).^23,24,33–36^ Diagnostic criteria for SJS/TEN and SOC and definition of CM of each study are summarized in Table 2 and Supplemental Table 1. The included studies identified *HLA* genotypes using polymerase chain reaction assays were followed by hybridization with sequence-specific oligonucleotide probes using commercially available bead-based typing kits (Wakunaga Pharmaceutical).^23,24,33–36^ No study reported sample-size calculations before recruiting patients, nor HWE information. A mean quality assessment using NOS of case control studies was 5.33 (range 4–7) (Supplemental Table 1).

**Table 2:** Case–control descriptions of included studies.

Author (year)	CM		Definition of SJS/TEN with SOC	Case	Control
	Definition of CM	Ascertainment method to identify causative drugs		Diagnostic Criteria	Diagnostic Criteria
Ueta et al. (2014)^23^	CM defined as NSAIDs and multi-ingredient	NR	Group1 KPUM: The diagnosis of SJS/TEN with SOC was based on a confirmed history of acute-onset high fever, serious mucocutaneous illness with skin eruptions, and the involvement of at least 2 mucosal sites including the oral cavity and ocular surface Group2 NIHS: The diagnosis of SJS/TEN was based on Bastuji-Garin et al.^a^	Group1 KPUM: Patients who had taken CM such as NSAIDs and multi-ingredient CM for a few, several days before disease onset for common-cold symptoms Group2 NIHS: Patients with newly-developed SJS/TEN	Healthy volunteers
Ueta et al. (2014)^24^	CM defined as multi-ingredient CM and NSAIDs	NR	The diagnosis of SJS/TEN based on a confirmed history of acute-onset high fever, serious mucocutaneous illness with skin eruptions, and involvement of at least two mucosal sites including the ocular surface. Acute stage of SOC were defined as patients who manifested a pseudomembrane and an epithelial defect on the ocular surface. Chronic stage of SOC were defined as patients who sequelae such as dry eye, trichiasis, symblepharon, and conjunctival invasion into the cornea	NR	Healthy volunteers
		NR			
		NR			
Kannabiran et al. (2017)^33^	NR	NR	The diagnosis of SJS/TEN with SOC was based on a confirmed history of acute-onset high fever, serious mucocutaneous manifestations with skin eruptions, and the involvement of at least 2 mucosal sites, including the oral cavity and ocular surface in the acute stage. In the chronic stage there were the ocular previously reported manifestations such as vascularization, corneal scarring, conjunctival inversion to the cornea, keratinization, symblepharon, scarring of the palpebral conjunctiva, trichiasis, and severe dry eye	NR	No history of SJS/TEN or related conditions or a history of cutaneous drug reactions
Wakamatsu, et al (2017)^34^	CM defined as Dipyrone and NSAIDs	NR	The diagnosis of SJS/TEN with SOCs was based on a confirmed history of acute-onset high fever, serious mucocutaneous illness with skin eruptions, and the involvement of at least 2 mucosal sites, including the oral cavity and ocular surface	Patients who had used CM for treatment of symptoms of common cold 1–14 days before disease onset	Healthy volunteers, including university employees and students, and patients who did not have any symptoms and signs similar to cases
Jongkhajornpong et al. (2018)^35^	CM defined as the drug that patients took for relieving cold symptoms, including NSAIDS, acetaminophen and other multi-ingredient CM	Causative agents were identified on the basis of documentation from dermatologists and immunologists	The diagnostic criteria of SJS/TEN were based on a confirmed history of acute onset of high fever, and skin eruption with at least two sites of serious mucocutaneous involvement including the oral mucosa and the ocular surface. SOC were defined as severe conjunctivitis pseudomembrane, and epithelial defect on the ocular surface in the acute stage and/or ocular sequelae such as dry eye, trichiasis, symblepharon and conjunctival invasion into the cornea in the chronic stage	Patients who were diagnosed with SJS/TEN either in acute, subacute or chronic phase between September 2014 and August 2017 in two university referral centers in Thailand, including Mahidol University (MU; Ramathibodi Hospital and Siriraj Hospital) and Chulalongkorn University (CU; King Chulalongkorn Memorial Hospital)	Healthy volunteers
Jun et al. (2019)^36^	NR	NR	The diagnostic criteria of SJS/TEN were based on history of acute-onset high fever, serious mucocutaneous illness with skin eruptions and involvement of at least two mucosal sites including the ocular surface. SOC were defined as pseudomembrane formation and an epithelial defect on the ocular surface in the acute stage, with ocular sequelae such as dry eye, trichiasis,symblepharon and conjunctival invasion into the cornea in the chronic stage	The patients who had SJS/TEN induced by CM such as NSAIDs during the first 3 months of exposure and was resolved after discontinuation	Without any known or previously diagnosed dermatological, allergic or systemic disease similar to SJS/TEN

*CM* cold medicine, *HLA* human leukocyte antigen, *KPUM* Kyoto Prefectural University of Medicine, *NIHS* National Institute of Health Sciences, *NR* not report, *NSAIDS* non-steroidal anti-inflammatory drugs, *SJS* Stevens–Johnson syndrome, *SOC* severe ocular complications, *TEN* toxic epidermal necrolysis.

^a^SJS defined as skin detachment below 10% of body surface area plus widespread macules or flat atypical targets; TEN defined as skin detachment detachment above 30% of the BSA plus widespread macules or flat atypical targets with spots with or without blisters or as detachment above 10% of body surface area with large epidermal sheets and without any macule or target without spots.^47^.

### Data analysis

The associations between *HLA* genotypes and CM-induced SJS/TEN with SOC of the included studies are summarized in Supplemental Table 2.

### *HLA-A* genotypes and CM-induced SJS/TEN with SOC

**Figure 2:**
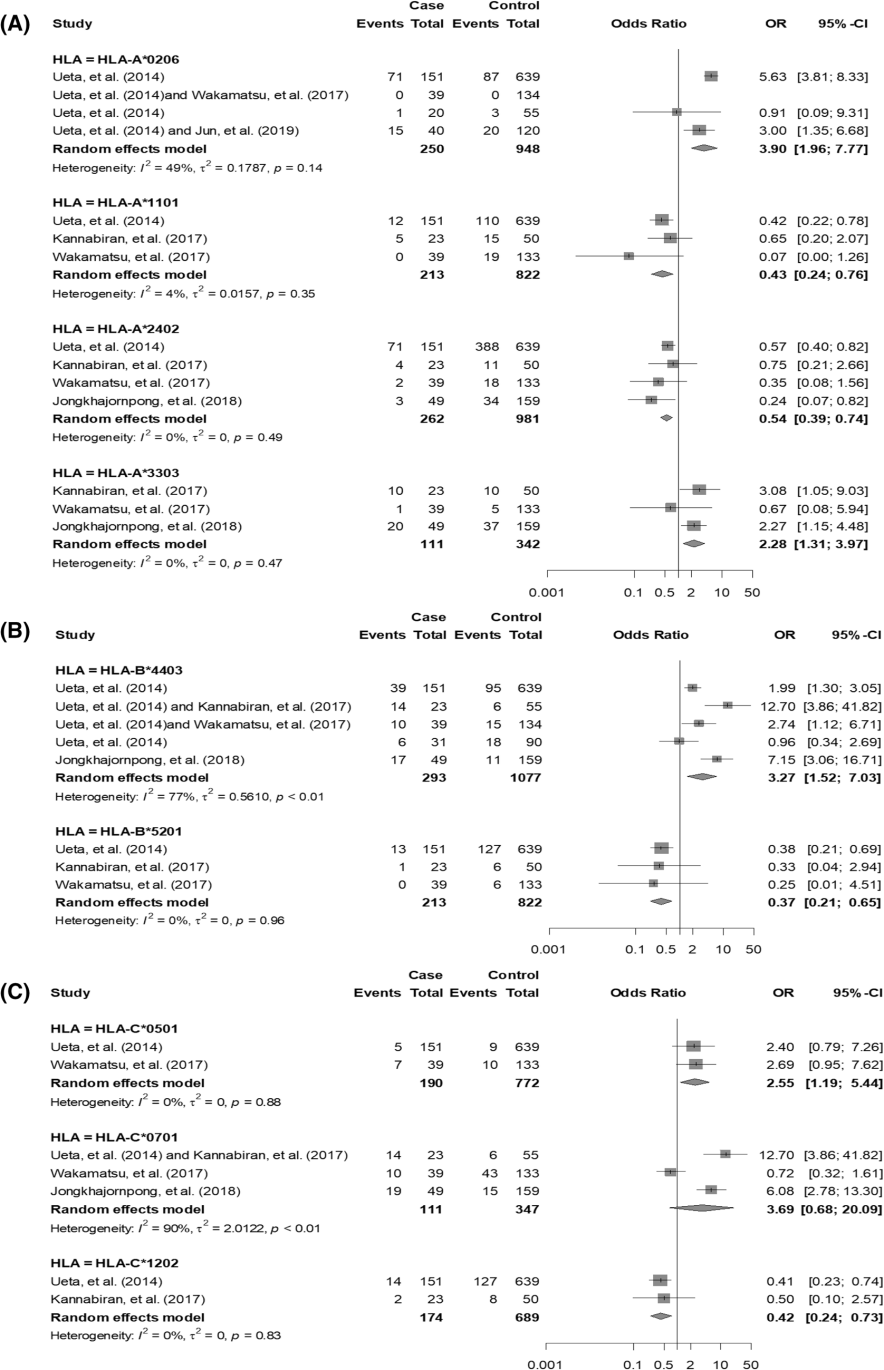
Forest plots of statistically significant associations between *HLA-A* genotypes and CM-induced SJS/TEN with SOC (**A**), statistically significant associations between *HLA-B* genotypes and CM-induced SJS/TEN with SOC (**B**), statistically significant associations between *HLA-C* genotypes and CM-induced SJS/TEN with SOC genotypes and CM-induced SJS/TEN with SOC.

All of the included studies investigated associations between 22 different *HLA-A* genotypes and CM-induced SJS/TEN with SOC.^23,24,33–36^ There were sufficient data to assess the associations between 9 different *HLA-A* genotypes and CM-induced SJS/TEN with SOC (i.e., HLA-A*0101^33,34^, HLA-A*0206^23,24,34,36^, HLA-A*0301^23,33,34^, HLA-A*1101^23,33,34^, HLA-A*2402^23,33–35^, HLA-A*2601^33,34^, HLA-A*3201^33,34^, HLA-A*3303^33–35^ and HLA-A*6801^33,34^) (Supplemental Table 2).Among these meta-analyses, we found statistically significant associations between HLA-A*0206, HLA-A*1101, HLA-A*2402, HLA-A*3303 and CM-induced SJS/TEN with SOC (Fig. 2 and Supplemental Table 2).

There were 3 studies investigating an association between HLA-A*0206 and CM-induced SJS/TEN with SOC in Japanese, Brazilian, Indian and Korean populations.^23,24,34,36^ The numbers of subjects carrying at least one allele of HLA-A*0206 were 87 out of 250 in cases and 110 out of 948 in controls. The overall OR was 3.90 (95% CI = 1.96–7.77). A moderate degree of heterogeneity across studies was observed in our analyses (*I*^2^ = 49.2%, *p* = 0.139) (Fig. 2 and Supplemental Table 2). Notably, an association between HLA-A*0206 and acetaminophen-induced SJS/TEN with SOC in Japanese were reported in Ueta et al.^23^ The overall OR was 6.52 (95% CI = 9.91–10.88).

There were 3 studies investigating an association between HLA-A*1101 and CM-induced SJS/TEN with SOC in Japanese, Brazilian and Indian populations.^23,33,34^ The numbers of subjects carrying at least one allele of HLA-A*1101 were 17 out of 213 in cases and 144 out of 822 in controls.^23,33,34^ The overall OR was 0.43 (95% CI = 0.24–0.76). There was no observed heterogeneity (*I*^2^ = 3.9%, *p* = 0.353) (Fig. 2 and Supplemental Table 2).

There were 4 studies studying an association between HLA-A*2402 and CM-induced SJS/TEN with SOC in Japanese, Brazilian, Indian and Thai populations.^13,22–24^ The numbers of subjects carrying at least one allele of HLA-A*2402 were 80 out of 262 in cases and 451 out of 981 in controls. The overall OR was 0.54 (95% CI = 0.39–0.74). There was no observed heterogeneity (*I*^2^ = 0.0%, *p* = 0.490) (Fig. 2 and Supplemental Table 2).

There were 3 studies investigating an association between HLA-A*3303 and CM-induced SJS/TEN with SOC in Brazilian, Indian and Thai populations.^33–35^ The numbers of subjects carrying at least one allele of HLA-A*3303 were 31 out of 111 in cases and 52 out of 342 in controls. The overall OR was 2.28 (95% CI = 1.31–3.97). There was no observed heterogeneity (*I*^2^ = 0.0%, *p* = 0.471) (Fig. 2 and Supplemental Table 2).

Nonetheless, an association between HLA-A*6601 and CM-induced SJS/TEN with SOC in Brazilian population was observed in Wakamatsu et al.^34^ The numbers of subjects carrying at least one allele of HLA-A*6601 were 6 out of 39 in cases and 1 out of 133 in controls. The overall OR was 24.00 (95% CI = 2.79–206) (Supplemental Table 2).

### *HLA-B* genotypes and CM-induced SJS/TEN with SOC

All of the included studies investigated associations between 40 different *HLA-B* genotypes and CM-induced SJS/TEN with SOC.^23,24,33–36^ There was only sufficient information to assess the associations between 8 different *HLA-B* genotypes and CM-induced SJS/TEN with SOC were performed meta-analyse (i.e., HLA-B*1501^23,34^, HLA-B*3501^33,34^, HLA-B*3503^33,34^, HLA-B*4402^23,34^, HLA-B*4403^23,24,33–35^, HLA-B*5101^33,34^, HLA-B*5201^23,33,34^ and HLA-B*5701^33,34^) (Supplemental Table 2). Among these meta-analyses, statistically significant associations between HLA-B*4403, HLA-B*5201 and CM-induced SJS/TEN with SOC were identified (Fig. 2 and Supplemental Table 2).

There were 3 studies investigating an association between HLA-B*4403 and CM-induced SJS/TEN with SOC in Japanese, Brazilian, Indian, Korean and Thai populations.^23,24,33–35^ The numbers of subjects carrying at least one allele of HLA-B*4403 were 86 out of 293 in cases and 145 out of 1,077 in controls. The overall OR was 3.27 (95% CI = 1.52–7.03). A high degree of heterogeneity across studies was found in our analyses (*I*^2^ = 76.9%, *p* = 0.002) (Fig. 2 and Supplemental Table 2). Interestingly, an association between HLA-B*4403 and acetaminophen-induced SJS/TEN with SOC in Japanese were reported in Ueta et al.^23^ The overall OR was 2.16 (95% CI = 1.27–3.78).

There were 3 studies investigating an association between HLA-B*5201 and CM-induced SJS/TEN with SOC in Japanese and Brazilian populations.^23,33,34^ The numbers of subjects carrying at least one allele of HLA-B*5201 were 14 out of 213 in cases and 139 out of 822 in controls. The overall OR was 0.37 (95% CI = 0.21–0.65). There was no observed heterogeneity (*I*^2^ = 0.0%, *p* = 0.956) (Fig. 2 and Supplemental Table 2).

An association between HLA-B*1301 and CM-induced SJS/TEN with SOC in Japanese population was observed in Ueta et al.^23^ The numbers of subjects carrying at least one allele of HLA-B*1301 were 12 out of 151 in cases and 19 out of 639 in controls. The overall OR was 2.82 (95% CI = 1.34–5.94) (Supplemental Table 2).

An association between HLA-B*1502 and CM-induced SJS/TEN with SOC in Indian population was reported in Kannabiran et al.^33^ The numbers of subjects carrying at least one allele of HLA-B*1502 were 4 out of 23 in cases and 1 out of 50 in controls. The overall OR was 10.32 (95% CI = 1.08–98.31) (Supplemental Table 2).

An association between HLA-B*4601 and CM-induced SJS/TEN with SOC in Japanese population was observed in Ueta et al.^23^ The numbers of subjects carrying at least one allele of HLA-B*4601 were 24 out of 151 in cases and 56 out of 639 in controls (ref). The overall OR was 1.97 (95% CI = 1.18–3.29) (Supplemental Table 2).

### ***HLA-C*** genotypes and CM-induced SJS/TEN with SOC

All of the included studies investigated associations between 19 different *HLA-C* genotypes and CM-induced SJS/TEN with SOC. There were sufficient data to assess the associations between 10 different *HLA-C* genotypes and CM-induced SJS/TEN with SOC (i.e., HLA-C*0102^33,34^, HLA-C*0303^34,36^, HLA-C*0304^23,34,36^, HLA-C*0401^33,34^, HLA-C*0501^13,23^, HLA-C*0602^33,34^, HLA-C*0701^24,33–35^, HLA-C*0702^33,34^, HLA-C*1202^13,22^ and HLA-C*1502^33,34^) (Supplement 2). Among these meta-analyses, we found statistically significant associations between HLA-C*0501, HLA-C*1202 and CM-induced SJS/TEN with SOC (Fig. 2 and Supplemental Table 2).

There were 2 studies reporting an association between HLA-C*0501 and CM-induced SJS/TEN with SOC in Brazilian and Indian populations.^23,34^ The numbers of subjects carrying at least one allele of HLA-C*0501 were 12 out of 190 in cases and 19 out of 772 in controls. The overall OR was 2.55 (95% CI = 1.19–5.44). There was no observed heterogeneity (*I*^2^ = 0.0%, *p* = 0.882) (Fig. 2 and Supplemental Table 2).

There were 2 studies investigating an association between HLA-C*1202 and CM-induced SJS/TEN with SOC in Japanese, Brazilian and Indian populations.^23,33^ The numbers of subjects carrying at least one allele of HLA-C*1202 were 16 out of 174 in cases and 135 out of 689 in controls. The overall OR was 0.42 (95% CI = 0.24–0.73). There was no observed heterogeneity (*I*^2^ = 0.0%, *p* = 0.827) (Fig. 2 and Supplemental Table 2).

An association between HLA-C*0801 and CM-induced SJS/TEN with SOC in Indian population was observed in Kannabiran et al.^33^ The numbers of subjects carrying at least one allele of HLA-C*0801 were 4 out of 23 in cases and 1 out of 50 in controls. The overall OR was 10.32 (95% CI = 1.08–98.31) (Supplemental Table 2).

An association between HLA-C*1203 and CM-induced SJS/TEN with SOC in Brazilian population was observed in Wakamatsu et al.^34^ The numbers of subjects carrying at least one allele of HLA-C*1203 were 7 out of 39 in cases and 5 out of 133 in controls. The overall OR was 5.60 (95% CI = 1.67–18.80) (Supplemental Table 2).

### *HLA* genotypes and CM-induced SJS/TEN

Based on the numbers of subjects carrying HLA-A*0206 and HLA-B*4403 in CM-induced SJS/TEN provided by Ueta et al.^23^ associations between HLA-A*0206, HLA-B*4403 and CM-induced SJS/TEN were not statistically significant. (OR = 0.91; 95% CI = 0.20–4.06 and OR = 0.17; 95% CI = 0.01–2.90, respectively).

## Discussion

To our knowledge, this is the first systematic review and meta-analysis study to identify the associations between *HLA* genotypes and CM-induced SJS/TEN with SOC. We found the associations between 81 different *HLA* genotypes and CM-induced SJS/TEN with SOC. Among these HLA genotypes, further meta-analysis could only be performed for 27 (Supplemental Table 2). HLA-A*0206, HLA-A*3303, HLA-B*4403 and HLA-C*0501 were identified as risks of CM-induced SJS/TEN with SOC (Fig. 2 and Supplemental Table 2). The heterogeneity across studies were identified in the association between HLA-A*0206, HLA-B*4403 and CM-induced SJS/TEN with SOC. These heterogeneities may be due to prevalence of susceptibility *HLA* genotypes in each ethnicity.

The heterogeneity across studies were identified in the association between HLA-A*0206, HLA-B*4403 and CM-induced SJS/TEN with SOC. These heterogeneities may be due to prevalence of susceptibility *HLA* genotypes in each ethnicity. The prevalence of HLA-A*0206 (ƒ) in Brazilian, Indian and Japanese were 0, 0.156–0.077, and 0.077–0.200, respectively. Whereas, the prevalence of HLA-B*4403 in Brazilian, Indian and Japanese were 0–0.053, 0–0.106, 0–0.122 and 0.042, respectively. However, the information concerning the gene allele frequencies among the Korean population is not available.^37^.

Interestingly, the associations between HLA-C*0304 and HLA-C*0701 and CM-induced SJS/TEN with SOC were not statistically significant in our pooling analyses (Supplement 2). However, HLA-C*0304 and HLA-C*0701 were associated with CM-induced SJS/TEN with SOC in some populations. Therefore, the risk of HLA-C*0304 and HLA-C*0701 and CM-induced SJS/TEN with SOC in these populations should be investigated, especially the associations between HLA-C*0701 and CM-induced SJS/TEN in Indian and Thai populations given the high odds ratios (Supplemental Table 2).

From our systematic review, HLA-A*6601, HLA-C*1203 were associated with CM-induced SJS/TEN with SOC in the Brazilian population.^34^ HLA-B*1301, HLA-B*4601 were associated with CM-induced SJS/TEN with SOC in Japanese population.^23^ HLA-B*1502, HLA-C*0801 were associated with CM-induced SJS/TEN with SOC in Indian population.^33^ Due to limited number of studies, more studies investigating associations between these *HLA* genotypes and CM-induced SJS/TEN with SOC in the same or different ethnicities are needed*.*

Since most of the included studies defined CM as multi-ingredient of CM including NSAIDs, an appropriate subgroup analysis was not possible. Therefore, the association between identified risk *HLA* genotypes and specific CMs for SJS/TEN with SOC within multi-ingredient formulations will require clarification. The associations between HLA-A*0206, HLA-B*4403 and acetaminophen-induced SJS/TEN with SOC in Japanese population were reported in Ueta et al.^23^ Therefore, future epidemiology studies identify the risk of *HLA* genotypes and other distinct CM (i.e. ibuprofen) induced SJS/TEN with SOC may be necessary. In addition, in silico studies to evaluate binding affinity between the *HLA* genotypes and these drugs or CM constituents to be more general as binding modelling for all potential antigens would be useful.^38,39^.

Since CM are categorized as over the counter drugs, they are easily accessible by the public. Therefore, prevalence of SJS/TEN caused by CM is likely higher than that caused by other prescription-controlled drugs.^36^ However, based on limited of information from the included studies, the associations between HLA-A*0206, HLA-B*4403 and CM-induced SJS/TEN were not identified in our analysis.

Within in silico studies, some CM (i.e., acetaminophen, ibuprofen, loxoprofen and ethenzamide) showed high binding affinities to peptide-binding groove of HLA-A*0206^40^ These high-affinity specific bindings of the suspected agents at their specific binding site within the HLA protein molecule may trigger molecular cascades contributing to the SJS/TEN with SOC. With this in silico molecular docking approach, these results might explain a possible mechanism contributing to SJS/TEN with or without SOC. However, some viral or microbial infections might be an additional factor contributing to develop CM-induced SJS/TEN with SOC. To further investigate which CMs’ ingredient(s) is responsible for this adverse drug reaction, a well-designed case–control study investigating an association between specific drugs (e.g. acetaminophen, ibuprofen) and certain *HLA genes* (e.g. HLA-A*0206, HLA-B*4403) should be conducted. Ueta et al.^41^ and Jongkhajornpong et al.^35^ hypothesized that patients who have genetic background with SJS/TEN with SOC are infected with some viruses or bacteria. These patients could develop an abnormal immune response. Whereas, CM such as NSAIDs can suppress production of prostaglandin E2 which downregulates, and this might augment with the abnormal immune response from the infections, resulting to develop SJS/TEN with SOC.^42^.

The associations between *HLA* genotypes and adverse drug reactions were documented in several studies.^15,19,43,44^ Interestingly, clinical utility of *HLA* genotypes screening prior to drugs use are limited due to low positive predictive value and higher negative predictive value.^43,45^ These information suggested that *HLA* genotypes may plays an important role in adverse drug reactions. However, combination of other genes, gene–gene interactions and environmental factors are possible resulting in these outcomes.^23,46^.

With our results demonstrating a significant association between using of CMs and the severe ADR, a genetic testing can be helpful. However, the CMs are commonly used as an over-the-counter drug in practically almost of people in populations worldwide, this will certainly include a large number of patients with limited numbers of cases. Consequently, the genetic screening prior to use of the CMs in general population might not be cost-effective. Nonetheless, for people with a family history of developing the ADRs with a possible involvement of CMs, a genetic screening may be beneficial.

## Conclusion

Statistically significant associations between HLA-A*0206, HLA-A*3303, HLA-B*4403, HLA-C*0501 and CM-induced SJS/TEN with SOC were identified. Thus, for patients’ safety, genetic screening among the populations at risk may be beneficial as well as changing labeling of the CM to increase awareness of the potential risk of developing SJS/TEN with SOC.

## Supplementary information

Supplementary file

## Abbreviations

CI: Confidence intervals
CM: Cold medicine
CM-induced SJS/TEN with SOC: Cold medicines-induced Stevens–Johnson syndrome/toxic epidermal necrolysis with severe ocular complications
HLAs: Human leukocyte antigens
HWE: Hardy–Weinberg equilibrium
NOS: The Newcastle–Ottawa scale
NSAIDS: Non-steroidal anti-inflammatory drugs
OR: Odds ratio
PCR: Polymerase chain reaction
SJS: Stevens–Johnson syndrome
SOC: Severe ocular complications
TEN: Toxic epidermal necrolysis
