# Supplementary material for: HLA genotypes and cold medicine-induced Stevens–Johnson syndrome/toxic epidermal necrolysis with severe ocular complications: a systematic review and meta-analysis
Source: Sci Rep. 2020 Jun 29;10:10589. doi: 10.1038/s41598-020-67610-5 (PMC7324363; doi:10.1038/s41598-020-67610-5)
Supplement: Supplementary file 1 — Supplementary file [file 41598_2020_67610_MOESM1_ESM.docx]

***HLA* genotypes and cold medicine-induced Stevens-Johnson syndrome/Toxic epidermal necrolysis with severe ocular complications: A systematic review and meta-analysis**

**Running title:** *HLA* genotypes and cold medicine-induced SJS/TEN with severe ocular complications

Wimonchat Tangamornsuksan, PhD ^1^

Sirikan Chanprasert, DDS, MSc ^2,3^

Phenphechaya Nadee, BSc ^3,4^

Saowalak Rungruang, BSc ^3,4^

Natnaree Meesilsat, BSc ^3^

Mayumi Ueta, MD, PhD ^5^

Manupat Lohitnavy, PhD* ^6, 7, 8^

1 Faculty of Medicine and Public Health, HRH Princess Chulabhorn College of Medical Science, Chulabhorn Royal Academy, Bangkok, Thailand

2 Department of Dental Public Health, Sirindhorn College of Public Health, Phitsanulok, Thailand

3 Integrative Cardiovascular Research Unit, Faculty of Medical Science, Naresuan University, Phitsanulok, Thailand

4 Department of Physiology, Faculty of Medical Science, Naresuan University, Phitsanulok, Thailand

5 Department of Frontier Medical Science and Technology for Ophthalmology, Kyoto Prefectural University of Medicine, Kyoto, Japan

6 Center of Excellence for Environmental Health & Toxicology, Faculty of Pharmaceutical Sciences, Naresuan University, Phitsanulok, Thailand

7 Pharmacokinetic Research Unit, Faculty of Pharmaceutical Sciences, Naresuan University, Phitsanulok, Thailand

8 Department of Pharmacy Practice, Faculty of Pharmaceutical Sciences, Naresuan University, Phitsanulok, Thailand

*Corresponding author:

Manupat Lohitnavy, Ph.D.

Associate Professor and Director

Center of Excellence for Environmental Health & Toxicology, Faculty of Pharmaceutical Sciences, Naresuan University, Phitsanulok, 65000, Thailand

Phone number (office): 66-5596-1888

E-mail: [manupatl@gmail.com](mailto:manupatl@gmail.com)

# Supplemental Table 1

**Case-control descriptions of included studies**

| **Author (Year)** | **CM** | | | **Definition of SJS/TEN with SOC** | **Case** | | **Control** | | **NOS rating** ^a^ |
| --- | --- | --- | --- | --- | --- | --- | --- | --- | --- |
|  | **Definition of CM** | **Suspected as causative drug (n)** | **Ascertainment method to identify causative drugs** |  | **Diagnostic Criteria** | **Ascertainment method (SJS/TEN with SOC evaluation)** | **Diagnostic Criteria** | **Ascertainment method** |  |
| Ueta, et al. (2014)[^23^](#_ENREF_23) | CM defined as NSAIDs and multi-ingredient | NR | NR | **Group1 KPUM:** The diagnosis of SJS/TEN with SOC was based on a confirmed history of acute-onset high fever, serious mucocutaneous illness with skin eruptions, and the involvement of at least 2 mucosal sites including the oral cavity and ocular surface.  **Group2 NIHS:** The diagnosis of SJS/TEN was based on Bastuji-Garin et al.[^43^](#_ENREF_43)^b^ | **Group1 KPUM:** Patients who had taken CM such as NSAIDs and multi-ingredient CM for a few, several days before disease onset for common-cold symptoms  **Group2 NIHS:** Patients with newly-developed SJS/TEN | NR | Healthy volunteers | NR | 5 |
| Ueta, et al. (2014)[^24^](#_ENREF_24) | CM defined as multi-ingredient CM and NSAIDs | **Brazilian (Ethnicity: Pardo, n=19; White, n=15; Black, n=3; White/Indian, n=1; White/Black/ Indian, n=1)**   - Aspirin  (n=2: Pardo, n=2) - Diclofenac  (n=6: Pardo, n=4; White, n=2) - Diclofenac and paracetamol  (n=1: Pardo, n=1) - Dipyrone  (n=25: Pardo, n=9; White, n=11; Black, n=3; White/Indian, n=1; White/Black/Indian, n=1) - Dipyrone and diclofenac  (n=1: Pardo=1) - Fenton (cold medicine)  (n=1: White, n=1) - Nimesulide  (n=1: Pardo, n=1) - Piroxicam  (n=1: White, n=1) - Tylenol (cold medicine)  (n=1: Pardo, n=1) | NR | The diagnosis of SJS/TEN based on a confirmed history of acute-onset high fever, serious mucocutaneous illness with skin eruptions, and involvement of at least two mucosal sites including the ocular surface. Acute stage of SOC were defined as patients who manifested a pseudomembrane and an epithelial defect on the ocular surface. Chronic stage of SOC were defined as patients who sequelae such as dry eye, trichiasis, symblepharon, and conjunctival invasion into the cornea | NR | Ophthalmologist | Healthy volunteers | NR | 4 |
|  |  | **Indian population (n=20)**   - Acetaminophen  (n=1) - Dicrofenac (n=1) - Ibuprofen (n=1) - Medicine for cold (NSAID) (n=1) - Medicine for cold (detail unknown) (n=13) - Mefenamic acid (n=1) - Medicine for cold (NSAID) (n=1) - Metamizole (n=1) | NR |  |  |  |  |  |  |
|  |  | **Korean (n=31)**   - Acetaminophen  (n=2) - Cold remedies (unknown) (n=10) - Ibuprofen (n=4) - Medicine for cold (detail unknown) (n=9) - NSAIDs (drug name unknown) (n=5) - Tylenol (cold medicine) (n=1) | NR |  |  |  |  |  |  |
| Kannabiran, et al. (2017)[^33^](#_ENREF_33) | NR | NR | NR | The diagnosis of SJS/TEN with SOC was based on a confirmed history of acute-onset high fever, serious mucocutaneous manifestations with skin eruptions, and the involvement of at least 2 mucosal sites, including the oral cavity and ocular surface in the acute stage. In the chronic stage there were the ocular previously reported manifestations such as vascularization, corneal scarring, conjunctival inversion to the cornea, keratinization, symblepharon, scarring of the palpebral conjunctiva, trichiasis, and severe dry eye. | NR | NR | No history of SJS/TEN  or related conditions or a history of cutaneous drug reactions | NR | 5 |
| Wakamatsu, et al.  (2017)[^34^](#_ENREF_34) | CM defined as Dipyrone and NSAIDs | - Dipyrone (n=37) - Acetaminophen (n=4) - Nimesulide (n=1) - Aspirin (n=4) - Diclofenac (n=7) - Piroxicam (n=1) - Codeine (n=1) | NR | The diagnosis of SJS/TEN with SOCs was based on a confirmed history of acute-onset high fever, serious mucocutaneous illness with skin eruptions, and the involvement of at least 2 mucosal sites, including the oral cavity and ocular surface | Patients who had used CM for treatment of symptoms of common cold 1 to 14 days before disease onset | NR | Healthy volunteers, including university  employees and students, and patients who did not have any symptoms and signs similar to cases | Question naire | 7 |
| Jongkhajornpong, et al. (2018)[^35^](#_ENREF_35) | CM defined as the drug that patients took for relieving cold symptoms, including non-steroidal anti-inflammatory  drugs (NSAIDS), acetaminophen and other multi-ingredient CM. | - Acetaminophen (n=18) - Combination of both acetaminophen and NSAIDs (n=1) - Multi-ingredient CM (n=4) - NSAIDs (n=10) - Unspecified CM (n=17) | Causative agents were identified  on the basis of documentation from dermatologists and immunologists | The diagnostic criteria of SJS/TEN were based on a confirmed history of acute onset of high fever, and skin eruption with at least two sites of serious mucocutaneous involvement including the oral mucosa and the ocular surface. SOC were defined as severe conjunctivitis,  pseudomembrane, and epithelial defect on the ocular surface in the acute stage and/or ocular sequelae such as dry eye, trichiasis, symblepharon and conjunctival invasion into the cornea in the chronic stage. | Patients who were diagnosed with SJS/TEN either in acute, subacute or chronic phase between September 2014 and August 2017 in two university referral centers in Thailand, including Mahidol University (MU; Ramathibodi Hospital and Siriraj Hospital) and Chulalongkorn University (CU; King Chulalongkorn  Memorial Hospital) | NR | Healthy volunteers | NR | 4 |
| Jun, et al. (2019)[^36^](#_ENREF_36) | NR | - Acetaminophen (n=5) - NSAIDs (n=11) - Unspecified CM (n=24) | NR | The diagnostic criteria of SJS/TEN were based on history of acute-onset high fever, serious mucocutaneous illness with skin eruptions and involvement of at least two mucosal sites including the ocular surface. SOC were defined as pseudomembrane  formation and an epithelial defect on the ocular surface in the acute stage, with ocular sequelae such as dry eye, trichiasis,  symblepharon and conjunctival invasion into the cornea in the chronic stage | The patients who had SJS/TEN induced by CM such as NSAIDs during the first 3 months of exposure and was resolved after discontinuation. | SJS/TEN were confirmed by either a dermatologist or an internist, according to clinical morphology by Roujeau [^44^](#_ENREF_44)^c^.  SOC were confirmed by Ophthalmologists. | Without any known or previously diagnosed dermatological, allergic or systemic disease similar to SJS/TEN | Questionnaire | 7 |

Abbreviations: CM=Cold medicine; HLA=Human leukocyte antigen; KPUM=Kyoto Prefectural University of Medicine; NIHS=National Institute of Health Sciences; NOS=The Newcastle-Ottawa scale; NR=Not report; NSAIDS=Non-steroidal anti-inflammatory drugs; SJS=Stevens–Johnson syndrome; SOC=Severe ocular complications; TEN=Toxic epidermal necrolysis

Footnote: ^a^The Newcastle-Ottawa scale (NOS) is a tool commonly used for evaluating the quality of the selected studies based on 3 following domains. Those are 1) Selection (4 points), 2) Comparability (2 points) and 3) Exposure (4 points).[^28^](#_ENREF_28) The total points of an evaluation are 10 points (range = 0-10 points). Thus, the more points awarded to the selected studies suggest a better quality of the studies.

^b^ SJS defined as skin detachment below 10% of body surface area plus widespread macules or flat atypical targets; TEN defined as skin detachment detachment above 30% of the BSA plus widespread macules or flat atypical targets with spots with or without blisters or as detachment above 10% of body surface area with large epidermal sheets and without any macule or target without spots.[^43^](#_ENREF_43)

^c^ SJS characterized by small blisters arising on purple macules. Lesions are widespread and usually predominate on the trunk. Confluence of blisters on limited areas leads to detachment below 10% of the body surface area; TEN is characterized by the same lesions than SJS but with confluence of blisters leading to positive Nikolski sign and to detachment of large epidermal sheets on more than 30% of the body surface area.[^44^](#_ENREF_44)

# Supplemental Table 2

**Summary odds ratios of the included studies categorized by *HLA* genotypes**

| ***HLA* genotypes** | **Author (Year)** | **Ethnicity** | **Study design** | **Case** | | **Control** | | **Odds Ratio (95%CI)** |
| --- | --- | --- | --- | --- | --- | --- | --- | --- |
|  |  |  |  | ***HLA genotypes* positive** | **Total** | ***HLA genotypes* positive** | **Total** |  |
| ***HLA-A* genotypes** | | | | | | | | |
| *HLA-A*0101* | Kannabiran, et al. (2017)[^33^](#_ENREF_33) | Indian | Case control | 4 | 23 | 14 | 50 | 0.54 (0.16-1.86) |
|  | Wakamatsu, et al. (2017)[^34^](#_ENREF_34) | Brazilian | Case control | 2 | 39 | 20 | 133 | 0.31 (0.07-1.37) |
|  | Subtotal (*I^2^*= 0.0%, *p*=0.561) | | | 6 | 62 | 34 | 183 | 0.43 (0.17-1.12) |
| *HLA-A*0201* | Wakamatsu, et al. (2017)[^34^](#_ENREF_34) | Brazilian | Case control | 18 | 39 | 61 | 133 | 1.01 (0.49-2.07) |
| *HLA-A*0202* | Wakamatsu, et al. (2017)[^34^](#_ENREF_34) | Brazilian | Case control | 1 | 39 | 5 | 133 | 0.67 (0.08-5.94) |
| *HLA-A*0205* | Wakamatsu, et al. (2017)[^34^](#_ENREF_34) | Brazilian | Case control | 3 | 39 | 3 | 133 | 3.61 (0.70-18.66) |
| ***HLA-A*0206*** | Ueta, et al. (2014)[^23^](#_ENREF_23) | Japanese | Case control | 71 | 151 | 87 | 639 | 5.63 (3.81-8.33) |
|  | Ueta, et al. (2014)[^24^](#_ENREF_24) and Wakamatsu, et al. (2017)[^34^](#_ENREF_34)^a^ | Brazilian | Case control | 0 | 39 | 0 | 134 | N/A |
|  | Ueta, et al. (2014)[^24^](#_ENREF_24) | Indian | Case control | 1 | 20 | 3 | 55 | 0.91 (0.09-9.31) |
|  | Ueta, et al. (2014)[^24^](#_ENREF_24) and Jun, et al. (2019)[^36^](#_ENREF_36)^b^ | Korean | Case control | 15 | 40 | 20 | 120 | 3.00 (1.35-6.68) |
|  | **Subtotal (*I^2^*=49.2 %, *p*=0.139)** | | | **87** | **250** | **110** | **948** | **3.90 (1.96-7.77)** |
| *HLA-A*0211* | Kannabiran, et al. (2017)[^33^](#_ENREF_33) | Indian | Case control | 2 | 23 | 7 | 50 | 0.59 (0.11-3.06) |
| *HLA-A*0301* | Ueta, et al. (2014)[^23^](#_ENREF_23) | Japanese | Case control | 5 | 151 | 23 | 639 | 0.92 (0.34-2.45) |
|  | Kannabiran, et al. (2017)[^33^](#_ENREF_33) | Indian | Case control | 1 | 23 | 4 | 50 | 0.52 (0.06-4.96) |
|  | Wakamatsu, et al. (2017)[^34^](#_ENREF_34) | Brazilian | Case control | 8 | 39 | 16 | 133 | 1.89 (0.74-4.81) |
|  | Subtotal (*I^2^*= 0.0%, *p*=0.425) | | | 14 | 213 | 43 | 822 | 1.24 (0.65-2.37) |
| ***HLA-A*1101*** | Ueta, et al. (2014)[^23^](#_ENREF_23) | Japanese | Case control | 12 | 151 | 110 | 639 | 0.42 (0.22-0.78) |
|  | Kannabiran, et al. (2017)[^33^](#_ENREF_33) | Indian | Case control | 5 | 23 | 15 | 50 | 0.65 (0.20-2.07) |
|  | Wakamatsu, et al. (2017)[^34^](#_ENREF_34) | Brazilian | Case control | 0 | 39 | 19 | 133 | 0.07 (0.00-1.26) |
|  | **Subtotal (*I^2^*= 3.9%, *p*= 0.353)** | | | **17** | **213** | **144** | **822** | **0.43 (0.24-0.76)** |
| ***HLA-A*2402*** | Ueta, et al. (2014)[^23^](#_ENREF_23) | Japanese | Case control | 71 | 151 | 388 | 639 | 0.57 (0.40-0.82) |
|  | Kannabiran, et al. (2017)[^33^](#_ENREF_33) | Indian | Case control | 4 | 23 | 11 | 50 | 0.75 (0.21-2.66) |
|  | Wakamatsu, et al. (2017)[^34^](#_ENREF_34) | Brazilian | Case control | 2 | 39 | 18 | 133 | 0.35 (0.08-1.56) |
|  | Jongkhajornpong, et al. (2018)[^35^](#_ENREF_35) | Thai | Case control | 3 | 49 | 34 | 159 | 0.24 (0.07-0.82) |
|  | **Subtotal (*I^2^*= 0.0%, *p*=0.490)** | | | **80** | **262** | **451** | **981** | **0.54 (0.39-0.74)** |
| *HLA-A*2601* | Kannabiran, et al. (2017)[^33^](#_ENREF_33) | Indian | Case control | 1 | 23 | 8 | 50 | 0.24 (0.03-2.03) |
|  | Wakamatsu, et al. (2017)[^34^](#_ENREF_34) | Brazilian | Case control | 2 | 39 | 4 | 133 | 1.74 (0.31-9.90) |
|  | Subtotal (*I^2^*= 52.4%, *p*=0.147) | | | 3 | 62 | 12 | 183 | 0.71 (0.10-5.20) |
| *HLA-A*2902* | Wakamatsu, et al. (2017)[^34^](#_ENREF_34) | Brazilian | Case control | 4 | 39 | 12 | 133 | 1.15 (0.35-3.80) |
| *HLA-A*3001* | Wakamatsu, et al. (2017)[^34^](#_ENREF_34) | Brazilian | Case control | 3 | 39 | 7 | 133 | 1.50 (0.37-6.10) |
| *HLA-A*3002* | Wakamatsu, et al. (2017)[^34^](#_ENREF_34) | Brazilian | Case control | 2 | 39 | 4 | 133 | 1.74 (0.31-9.90) |
| *HLA-A*3101* | Wakamatsu, et al. (2017)[^34^](#_ENREF_34) | Brazilian | Case control | 4 | 39 | 13 | 133 | 1.06 (0.32-3.44) |
| *HLA-A*3201* | Kannabiran, et al. (2017)[^33^](#_ENREF_33) | Indian | Case control | 0 | 23 | 6 | 50 | 0.15 (0.01-2.70) |
|  | Wakamatsu, et al. (2017)[^34^](#_ENREF_34) | Brazilian | Case control | 1 | 39 | 5 | 133 | 0.68 (0.08-5.94) |
|  | Subtotal (*I^2^*= 0.0%, *p*=0.399) | | | 1 | 62 | 11 | 183 | 0.39 (0.07-2.23) |
| ***HLA-A*3303*** | Kannabiran, et al. (2017)[^33^](#_ENREF_33) | Indian | Case control | 10 | 23 | 10 | 50 | 3.08 (1.05-9.03) |
|  | Wakamatsu, et al. (2017)[^34^](#_ENREF_34) | Brazilian | Case control | 1 | 39 | 5 | 133 | 0.67 (0.08-5.94) |
|  | Jongkhajornpong, et al. (2018)[^35^](#_ENREF_35) | Thai | Case control | 20 | 49 | 37 | 159 | 2.27 (1.15-4.48) |
|  | **Subtotal (*I^2^*= 0.0%, *p*=0.471)** | | | **31** | **111** | **52** | **342** | **2.28 (1.31-3.97)** |
| *HLA-A*3402* | Wakamatsu, et al. (2017)[^34^](#_ENREF_34) | Brazilian | Case control | 2 | 39 | 3 | 133 | 2.34 (0.38-14.55) |
| *HLA-A*3601* | Wakamatsu, et al. (2017)[^34^](#_ENREF_34) | Brazilian | Case control | 2 | 39 | 7 | 133 | 0.97 (0.19-4.89) |
| ***HLA-A*6601*** | **Wakamatsu, et al. (2017)**[**^34^**](#_ENREF_34) | **Brazilian** | **Case control** | **6** | **39** | **1** | **133** | **24.00 (2.79-206.0)** |
| *HLA-A*6801* | Kannabiran, et al. (2017)[^33^](#_ENREF_33) | Indian | Case control | 5 | 23 | 5 | 50 | 2.50 (0.65-9.69) |
|  | Wakamatsu, et al. (2017)[^34^](#_ENREF_34) | Brazilian | Case control | 3 | 39 | 9 | 133 | 1.14 (0.30-4.47) |
|  | Subtotal (*I^2^*= 0.0%, *p*=0.427) | | | 8 | 62 | 14 | 183 | 1.70 (0.65-4.43) |
| *HLA-A*6802* | Wakamatsu, et al. (2017)[^34^](#_ENREF_34) | Brazilian | Case control | 2 | 39 | 6 | 133 | 1.14 (0.22-5.91) |
| *HLA-A*7401* | Wakamatsu, et al. (2017)[^34^](#_ENREF_34) | Brazilian | Case control | 2 | 39 | 6 | 133 | 1.14 (0.22-5.91) |
| ***HLA-B* genotypes** | | | | | | | | |
| *HLA-B*0702* | Wakamatsu, et al. (2017)[^34^](#_ENREF_34) | Brazilian | Case control | 1 | 39 | 17 | 133 | 0.18 (0.02-1.40) |
| *HLA-B*0705* | Kannabiran, et al. (2017)[^33^](#_ENREF_33) | Indian | Case control | 1 | 23 | 9 | 50 | 0.21 (0.03-1.74) |
| *HLA-B*0801* | Wakamatsu, et al. (2017)[^34^](#_ENREF_34) | Brazilian | Case control | 1 | 39 | 20 | 133 | 0.15 (0.02-1.15) |
| ***HLA-B*1301*** | **Ueta, et al. (2014)**[**^23^**](#_ENREF_23) | **Japanese** | **Case control** | **12** | **151** | **19** | **639** | **2.82 (1.34-5.94)** |
| *HLA-B*1302* | Wakamatsu, et al. (2017)[^34^](#_ENREF_34) | Brazilian | Case control | 1 | 39 | 3 | 133 | 1.14 (0.12-11.28) |
| *HLA-B*1401* | Wakamatsu, et al. (2017)[^34^](#_ENREF_34) | Brazilian | Case control | 0 | 39 | 4 | 133 | 0.36 (0.02-6.91) |
| *HLA-B*1501* | Ueta, et al. (2014)[^23^](#_ENREF_23) and Wakamatsu, et al. (2017)[^34^](#_ENREF_34)^a^ | Japanese | Case control | 13 | 51 | 108 | 639 | 1.68 (0.87-3.26) |
|  | Wakamatsu, et al. (2017)[^34^](#_ENREF_34) | Brazilian | Case control | 1 | 39 | 6 | 133 | 0.56 (0.07-4.77) |
|  | Subtotal (*I^2^*= 0.0%, *p*=0.332) | | | 14 | 90 | 114 | 772 | 1.53 (0.81-2.88) |
| ***HLA-B*1502*** | **Kannabiran, et al. (2017)**[**^33^**](#_ENREF_33) | **Indian** | **Case control** | **4** | **23** | **1** | **50** | **10.32 (1.08-98.31)** |
| *HLA-B*1510* | Wakamatsu, et al. (2017)[^34^](#_ENREF_34) | Brazilian | Case control | 1 | 39 | 4 | 133 | 0.85 (0.09-7.82) |
| *HLA-B*1801* | Wakamatsu, et al. (2017)[^34^](#_ENREF_34) | Brazilian | Case control | 3 | 39 | 7 | 133 | 1.50 (0.37-6.10) |
| *HLA-B*2704* | Jongkhajornpong, et al. (2018)[^35^](#_ENREF_35) | Thai | Case control | 0 | 49 | 15 | 159 | 0.09 (0.01-1.60) |
| *HLA-B*2705* | Wakamatsu, et al. (2017)[^34^](#_ENREF_34) | Brazilian | Case control | 2 | 39 | 3 | 133 | 2.34 (0.38-14.55) |
| *HLA-B*3501* | Kannabiran, et al. (2017)[^33^](#_ENREF_33) | Indian | Case control | 2 | 23 | 4 | 50 | 1.10 (0.19-6.46) |
|  | Wakamatsu, et al. (2017)[^34^](#_ENREF_34) | Brazilian | Case control | 4 | 39 | 18 | 133 | 0.73 (0.23-2.30) |
|  | Subtotal (*I^2^*= 0.0%, *p*=0.707) | | | 6 | 62 | 22 | 183 | 0.82 (0.31-2.16) |
| *HLA-B*3502* | Wakamatsu, et al. (2017)[^34^](#_ENREF_34) | Brazilian | Case control | 2 | 39 | 3 | 133 | 2.34 (0.38-14.55) |
| *HLA-B*3503* | Kannabiran, et al. (2017)[^33^](#_ENREF_33) | Indian | Case control | 4 | 23 | 6 | 50 | 1.54 (0.39-6.11) |
|  | Wakamatsu, et al. (2017)[^34^](#_ENREF_34) | Brazilian | Case control | 2 | 39 | 4 | 133 | 1.74 (0.31-9.90) |
|  | Subtotal (*I^2^*= 0.0%, *p*=0.914) | | | 6 | 62 | 10 | 183 | 1.62 (0.55-4.75) |
| *HLA-B*3508* | Wakamatsu, et al. (2017)[^34^](#_ENREF_34) | Brazilian | Case control | 0 | 39 | 4 | 133 | 0.36 (0.02-6.91) |
| *HLA-B*3801* | Wakamatsu, et al. (2017)[^34^](#_ENREF_34) | Brazilian | Case control | 3 | 39 | 2 | 133 | 5.46 (0.88-33.92) |
| *HLA-B*3903* | Wakamatsu, et al. (2017)[^34^](#_ENREF_34) | Brazilian | Case control | 1 | 39 | 4 | 133 | 0.85 (0.09-7.82) |
| *HLA-B*4001* | Wakamatsu, et al. (2017)[^34^](#_ENREF_34) | Brazilian | Case control | 1 | 39 | 3 | 133 | 1.14 (0.12-11.28) |
| *HLA-B*4002* | Wakamatsu, et al. (2017)[^34^](#_ENREF_34) | Brazilian | Case control | 2 | 39 | 3 | 133 | 2.34 (0.38-14.55) |
| *HLA-B*4006* | Kannabiran, et al. (2017)[^33^](#_ENREF_33) | Indian | Case control | 1 | 23 | 14 | 50 | 0.12 (0.01-0.95) |
| *HLA-B*4101* | Wakamatsu, et al. (2017)[^34^](#_ENREF_34) | Brazilian | Case control | 0 | 39 | 4 | 133 | 0.36 (0.02-6.91) |
| *HLA-B*4201* | Wakamatsu, et al. (2017)[^34^](#_ENREF_34) | Brazilian | Case control | 2 | 39 | 6 | 133 | 1.14 (0.22-5.91) |
| *HLA-B*4401* | Jongkhajornpong, et al. (2018)[^35^](#_ENREF_35) | Thai | Case control | 2 | 49 | 30 | 159 | 0.18 (0.04-0.80) |
| *HLA-B*4402* | Ueta, et al. (2014)[^23^](#_ENREF_23) | Japanese | Case control | 5 | 151 | 9 | 639 | 2.40 (0.79-7.26) |
|  | Wakamatsu, et al. (2017)[^34^](#_ENREF_34) | Brazilian | Case control | 6 | 39 | 12 | 133 | 1.83 (0.64-5.25) |
|  | Subtotal (*I^2^*= 0.0%, *p*=0.731) | | | 11 | 190 | 21 | 772 | 2.08 (0.97-4.47) |
| ***HLA-B*4403*** | Ueta, et al. (2014)[^23^](#_ENREF_23) | Japanese | Case control | 39 | 151 | 95 | 639 | 1.99 (1.30-3.05) |
|  | Ueta, et al. (2014)[^24^](#_ENREF_24) and Kannabiran, et al. (2017)[^33^](#_ENREF_33)^c^ | Indian | Case control | 14 | 23 | 6 | 55 | 12.70 (3.86-41.82) |
|  | Ueta, et al.(2014)[^24^](#_ENREF_24) and Wakamatsu, et al. (2017)[^34^](#_ENREF_34)^a^ | Brazilian | Case control | 10 | 39 | 15 | 134 | 2.74 (1.12-6.71) |
|  | Ueta, et al. (2014)[^24^](#_ENREF_24) | Korean | Case control | 6 | 31 | 18 | 90 | 0.96 (0.34-2.69) |
|  | Jongkhajornpong, et al. (2018)[^35^](#_ENREF_35) | Thai | Case control | 17 | 49 | 11 | 159 | 7.15 (3.06-16.70) |
|  | **Subtotal (*I^2^*= 76.9%, *p*=0.002)** | | | **86** | **293** | **145** | **1,077** | **3.27 (1.52-7.03)** |
| *HLA-B*4501* | Wakamatsu, et al. (2017)[^34^](#_ENREF_34) | Brazilian | Case control | 1 | 39 | 4 | 133 | 0.85 (0.09-7.82) |
| ***HLA-B*4601*** | **Ueta, et al. (2014)**[**^23^**](#_ENREF_23) | **Japanese** | **Case control** | **24** | **151** | **56** | **639** | **1.97 (1.18-3.29)** |
| *HLA-B*4901* | Wakamatsu, et al. (2017)[^34^](#_ENREF_34) | Brazilian | Case control | 3 | 39 | 9 | 133 | 1.15 (0.30-4.47) |
| *HLA-B*5001* | Wakamatsu, et al. (2017)[^34^](#_ENREF_34) | Brazilian | Case control | 2 | 39 | 2 | 133 | 3.54 (0.48-26.00) |
| *HLA-B*5101* | Kannabiran, et al. (2017)[^33^](#_ENREF_33) | Indian | Case control | 2 | 23 | 5 | 50 | 0.86 (0.15-4.79) |
|  | Wakamatsu, et al. (2017)[^34^](#_ENREF_34) | Brazilian | Case control | 2 | 39 | 25 | 133 | 0.23 (0.05-1.03) |
|  | Subtotal (*I^2^*= 22.6%, *p*=0.256) | | | 4 | 62 | 30 | 183 | 0.42 (0.12-1.50) |
| ***HLA-B*5201*** | Ueta, et al. (2014)[^23^](#_ENREF_23) | Japanese | Case control | 13 | 151 | 127 | 639 | 0.38 (0.21-0.69) |
|  | Kannabiran, et al. (2017)[^33^](#_ENREF_33) | Indian | Case control | 1 | 23 | 6 | 50 | 0.33 (0.04-2.94) |
|  | Wakamatsu, et al. (2017)[^34^](#_ENREF_34) | Brazilian | Case control | 0 | 39 | 6 | 133 | 0.25 (0.01-4.51) |
|  | **Subtotal (*I^2^*= 0.0%, *p*=0.956)** | | | 14 | 213 | 139 | 822 | **0.37 (0.21-0.65)** |
| *HLA-B*5301* | Wakamatsu, et al. (2017)[^34^](#_ENREF_34) | Brazilian | Case control | 4 | 39 | 12 | 133 | 1.15 (0.35-3.80) |
| *HLA-B*5401* | Ueta, et al. (2014)[^23^](#_ENREF_23) | Japanese | Case control | 15 | 151 | 94 | 639 | 0.64 (0.36-1.14) |
| *HLA-B*5501* | Wakamatsu, et al. (2017)[^34^](#_ENREF_34) | Brazilian | Case control | 1 | 39 | 4 | 133 | 0.85 (0.09-7.82) |
| *HLA-B*5601* | Wakamatsu, et al. (2017)[^34^](#_ENREF_34) | Brazilian | Case control | 0 | 39 | 4 | 133 | 0.36 (0.02-6.91) |
| *HLA-B*5701* | Kannabiran, et al. (2017)[^33^](#_ENREF_33) | Indian | Case control | 0 | 23 | 10 | 50 | 0.08 (0.01-1.47) |
|  | Wakamatsu, et al. (2017)[^34^](#_ENREF_34) | Brazilian | Case control | 2 | 39 | 8 | 133 | 0.85 (0.17-4.15) |
|  | Subtotal (*I^2^*= 54.0%, *p*=0.141) | | | 2 | 62 | 18 | 183 | 0.35 (0.03-3.68) |
| *HLA-B*5703* | Wakamatsu, et al. (2017)[^34^](#_ENREF_34) | Brazilian | Case control | 0 | 39 | 4 | 133 | 0.36 (0.02-6.91) |
| *HLA-B*5801* | Wakamatsu, et al. (2017)[^34^](#_ENREF_34) | Brazilian | Case control | 5 | 39 | 7 | 133 | 2.65 (0.79-8.86) |
| *HLA-B*5802* | Wakamatsu, et al. (2017)[^34^](#_ENREF_34) | Brazilian | Case control | 3 | 39 | 6 | 133 | 1.76 (0.42-7.40) |
| ***HLA-C* genotypes** | | | | | | | | |
| *HLA-C*0102* | Kannabiran, et al. (2017)[^33^](#_ENREF_33) | Indian | Case control | 0 | 23 | 5 | 50 | 0.18 (0.01-3.32) |
|  | Wakamatsu, et al. (2017)[^34^](#_ENREF_34) | Brazilian | Case control | 1 | 39 | 8 | 133 | 0.41 (0.05-3.39) |
|  | Subtotal (*I^2^*= 0.0%, *p*=0.644) | | | 1 | 62 | 13 | 183 | 0.31 (0.06-1.71) |
| *HLA-C*0202* | Wakamatsu, et al. (2017)[^34^](#_ENREF_34) | Brazilian | Case control | 4 | 39 | 10 | 133 | 1.41 (0.42-4.76) |
| *HLA-C*0302* | Wakamatsu, et al. (2017)[^34^](#_ENREF_34) | Brazilian | Case control | 1 | 39 | 6 | 133 | 0.56 (0.07-4.77) |
| *HLA-C*0303* | Wakamatsu, et al. (2017)[^34^](#_ENREF_34) | Brazilian | Case control | 2 | 39 | 11 | 133 | 0.60 (0.13-2.83) |
|  | Jun, et al. (2019)[^36^](#_ENREF_36)^b^ | Korean | Case control | 1 | 40 | 24 | 120 | 0.10 (0.01-0.79) |
|  | Subtotal (*I^2^*= 49.5%, *p*=0.159) | | | 3 | 79 | 35 | 253 | 0.28 (0.05-1.66) |
| *HLA-C*0304* | Ueta, et al. (2014)[^23^](#_ENREF_23) | Japanese | Case control | 48 | 151 | 141 | 639 | 1.65 (1.11-2.43) |
|  | Wakamatsu, et al. (2017)[^34^](#_ENREF_34) | Brazilian | Case control | 2 | 39 | 11 | 133 | 0.60 (0.13-2.83) |
|  | Ueta, et al. (2014)[^24^](#_ENREF_24) and Jun, et al. (2019)[^36^](#_ENREF_36)^b^ | Korean | Case control | 12 | 40 | 13 | 120 | 3.53 (1.45-8.57) |
|  | Subtotal (*I^2^*= 53.8%, *p*=0.115) | | | 62 | 230 | 165 | 892 | 1.79 (0.87-3.66) |
| *HLA-C*0401* | Kannabiran, et al. (2017)[^33^](#_ENREF_33) | Indian | Case control | 5 | 23 | 16 | 50 | 0.59 (0.19-1.87) |
|  | Wakamatsu, et al. (2017)[^34^](#_ENREF_34) | Brazilian | Case control | 15 | 39 | 41 | 133 | 1.40 (0.67-2.95) |
|  | Subtotal (*I^2^*= 34.5%, *p*=0.216) | | | 20 | 62 | 57 | 183 | 1.02 (0.45-2.32) |
| ***HLA-C*0501*** | Ueta, et al. (2014)[^23^](#_ENREF_23) | Japanese | Case control | 5 | 151 | 9 | 639 | 2.40 (0.79-7.26) |
|  | Wakamatsu, et al. (2017)[^34^](#_ENREF_34) | Brazilian | Case control | 7 | 39 | 10 | 133 | 2.69 (0.95-7.62) |
|  | **Subtotal (*I^2^*= 0.0%, *p*=0.882)** | | | **12** | **190** | **19** | **772** | **2.55 (1.19-5.44)** |
| *HLA-C*0602* | Kannabiran, et al. (2017)[^33^](#_ENREF_33) | Indian | Case control | 0 | 23 | 14 | 50 | 0.05 (0.00-0.94) |
|  | Wakamatsu, et al. (2017)[^34^](#_ENREF_34) | Brazilian | Case control | 9 | 39 | 15 | 133 | 2.36 (0.94-5.91) |
|  | Subtotal (*I^2^*= 87.5%, *p*=0.005) | | | 9 | 62 | 29 | 183 | 0.43 (0.01-29.61) |
| *HLA-C*0701* | Ueta, et al. (2014)[^24^](#_ENREF_24) and Kannabiran, et al. (2017)[^33^](#_ENREF_33)^c^ | Indian | Case control | 14 | 23 | 6 | 55 | 12.70 (3.86-41.82) |
|  | Wakamatsu, et al. (2017)[^34^](#_ENREF_34) | Brazilian | Case control | 10 | 39 | 43 | 133 | 0.72 (0.32-1.62) |
|  | Jongkhajornpong, et al. (2018)[^35^](#_ENREF_35) | Thai | Case control | 19 | 49 | 15 | 159 | 6.08 (2.78-13.30) |
|  | Subtotal (*I^2^*= 90.4%, *p*=0.000) | | | 43 | 111 | 64 | 347 | 3.69 (0.68-20.09) |
| *HLA-C*0702* | Kannabiran, et al. (2017)[^33^](#_ENREF_33) | Indian | Case control | 3 | 23 | 14 | 50 | 0.39 (0.10-1.51) |
|  | Wakamatsu, et al. (2017)[^34^](#_ENREF_34) | Brazilian | Case control | 5 | 39 | 25 | 133 | 0.64 (0.23-1.79) |
|  | Subtotal (*I^2^*= 0.0%, *p*=0.567) | | | 8 | 62 | 39 | 183 | 0.53 (0.23-1.21) |
| *HLA-C*0704* | Wakamatsu, et al. (2017)[^34^](#_ENREF_34) | Brazilian | Case control | 0 | 39 | 4 | 133 | 0.36 (0.02-6.91) |
| ***HLA-C*0801*** | **Kannabiran, et al. (2017)**[**^33^**](#_ENREF_33) | **Indian** | **Case control** | **4** | **23** | **1** | **50** | **10.32 (1.08-98.31)** |
| *HLA-C*0802* | Wakamatsu, et al. (2017)[^34^](#_ENREF_34) | Brazilian | Case control | 0 | 39 | 7 | 133 | 0.21 (0.01-3.82) |
| ***HLA-C*1202*** | Ueta, et al. (2014)[^23^](#_ENREF_23) | Japanese | Case control | 14 | 151 | 127 | 639 | 0.41 (0.23-0.74) |
|  | Kannabiran, et al. (2017)[^33^](#_ENREF_33) | Indian | Case control | 2 | 23 | 8 | 50 | 0.50 (0.10-2.57) |
|  | **Subtotal (*I^2^*= 0.0%, *p*=0.827)** | | | **16** | **174** | **135** | **689** | **0.42 (0.24-0.73)** |
| ***HLA-C*1203*** | **Wakamatsu, et al. (2017)**[**^34^**](#_ENREF_34) | **Brazilian** | **Case control** | **7** | **39** | **5** | **133** | **5.60 (1.67-18.80)** |
| *HLA-C*1402* | Wakamatsu, et al. (2017)[^34^](#_ENREF_34) | Brazilian | Case control | 0 | 39 | 6 | 133 | 0.25 (0.01-4.51) |
| *HLA-C*1502* | Kannabiran, et al. (2017)[^33^](#_ENREF_33) | Indian | Case control | 0 | 23 | 11 | 50 | 0.07 (0.00-1.30) |
|  | Wakamatsu, et al. (2017)[^34^](#_ENREF_34) | Brazilian | Case control | 2 | 39 | 13 | 133 | 0.50 (0.11-2.31) |
|  | Subtotal (*I^2^*= 30.8%, *p*=0.229) | | | 2 | 62 | 24 | 183 | 0.28 (0.05-1.69) |
| *HLA-C*1601* | Wakamatsu, et al. (2017)[^34^](#_ENREF_34) | Brazilian | Case control | 3 | 39 | 15 | 133 | 0.66 (0.18-2.39) |
| *HLA-C*1701* | Wakamatsu, et al. (2017)[^34^](#_ENREF_34) | Brazilian | Case control | 2 | 39 | 10 | 133 | 0.67 (0.14-3.17) |

Abbreviations: 95% CI = 95% confidence intervals; HLA = human leukocyte antigen

Footnote: ^a^ Ueta, et al. (2014)[^24^](#_ENREF_24) and Wakamatsu, et al. (2017)[^34^](#_ENREF_34) used the same population. However, the size of the population in Ueta, et al. (2014)[^24^](#_ENREF_24) was larger. Therefore population from Ueta, et al. (2014)[^24^](#_ENREF_24) were employed.

^b^ Ueta, et al. (2014)[^23^](#_ENREF_23) and Jun, et al. (2019)[^36^](#_ENREF_36) used the same population. However, the size of the population in Jun, et al. (2019)[^36^](#_ENREF_36) was larger. Therefore population from Jun, et al. (2019)[^36^](#_ENREF_36) were employed.

^c^ Ueta, et al. (2014)[^24^](#_ENREF_24) and Kannabiran, et al. (2017)[^33^](#_ENREF_33) used case from Kannabiran, et al whereas, controls was from Ueta, et al. (2014)[^24^](#_ENREF_24)
